# Severe distinct dysautonomia in *RFC1* ‐related disease associated with Parkinsonism

**DOI:** 10.1111/jns.12515

**Published:** 2022-10-07

**Authors:** Christopher J. Record, Rana Alnasser Alsukhni, Riccardo Curro, Diego Kaski, John S. Rubin, Huw R. Morris, Andrea Cortese, Valeria Iodice, Mary M. Reilly

**Affiliations:** ^1^ Department of Neuromuscular Diseases UCL Queen Square Institute of Neurology London UK; ^2^ Autonomic Unit National Hospital for Neurology and Neurosurgery Queen Square London UK; ^3^ Department of Brain and Behavioral Sciences University of Pavia Pavia Italy; ^4^ Department of Clinical and Movement Neurosciences UCL Queen Square Institute of Neurology London UK; ^5^ Royal National ENT and Eastman Dental Hospitals UCLH London UK; ^6^ Department of Targeted Intervention University College London London UK; ^7^ UCL Queen Square Institute of Neurology, Faculty of Brain Sciences University College London London UK

**Keywords:** autonomic, CANVAS, MSA, Parkinsons, RFC1

## Abstract

Biallelic repeat expansions in replication factor C subunit 1 (*RFC1*) have recently been found to cause cerebellar ataxia, neuropathy and vestibular areflexia syndrome (CANVAS). Additional features that have been described include Parkinsonism and a multiple system atrophy (MSA)‐like syndrome. CANVAS can include features of dysautonomia, but they are much milder than typically seen in MSA. We report a detailed autonomic phenotype of multisystem *RFC1*‐related disease presenting initially as CANVAS. Our patient presented aged 61 with a sensory ataxic neuropathy who rapidly developed widespread autonomic failure and Parkinsonism. The autonomic profile was of a mixed pre‐ and post‐ganglionic syndrome with progressive involvement of sympathetic and parasympathetic cardiovascular and sudomotor function. The Parkinsonism did not respond to levodopa. We present a patient with CANVAS and biallelic *RFC1* expansions who developed Parkinsonism with severe autonomic involvement similar to that seen in classical MSA. The link between MSA and CANVAS remains uncertain.

## 1 BACKGROUND AND AIMS

Biallelic pentanucleotide repeat expansions in the replication factor C subunit 1 (*RFC1*) gene have recently been found to cause cerebellar ataxia, neuropathy and vestibular areflexia syndrome (CANVAS). Chronic cough and dysautonomia are also frequently seen.^1^ Subsequently, cases mimicking Parkinsons disease and multiple system atrophy (MSA) type‐C have been reported, with variable responses to levodopa.^1^, ^2^, ^3^ However, there were no patients identified with pathogenic biallelic *RFC1* expansions in a cohort of 336 pathologically confirmed MSA cases, suggesting that *RFC1* expansions are not a common cause of MSA.^4^ Here, we present a case of *RFC1*‐related disease with Parkinsonism and an autonomic profile that resembles that typically seen in MSA.

## 2 CASE REPORT

### 2.1 Clinical presentation

A 61‐year‐old white British man was assessed, who had developed sensory symptoms in his 40s. He described a progressive loss of sensation starting in his feet, progressing to involve the upper limbs. His walking was unsteady and his hands clumsy, particularly in the dark. There was no weakness, but there was a year‐long history of bulbar symptoms including hoarseness and occasional choking. He had a chronic cough for decades. In his teens, he had had postural fainting episodes. Pre‐syncopal and syncopal episodes recurred aged 40, provoked by coughing, prolonged exercise, or standing. He also reported urinary urgency and nocturia. There was no vertigo or oscillopsia. He experienced vivid nightmares and his wife reported dream re‐enactment and “kicking‐out” in bed possibly suggestive of a REM sleep behaviour disorder. He was born of non‐consanguineous parents and one of seven siblings, at least two of whom had symptoms of sensory neuropathy and chronic cough. His parents were asymptomatic. Examination revealed a mildly ataxic gait. Rombergs sign was negative. He had non‐sustained gaze‐evoked nystagmus in the horizontal plane but a normal head impulse test. He had a mild postural tremor in the upper limbs but no pseudoathetosis. Tone and power testing were normal and there was a length‐dependent sensory deficit characterised by reduction of pinprick all over the body but worse distally, normal vibration in the upper limbs but absent to the costal margins bilaterally in the lower limbs and normal proprioception. Reflexes were present apart from absent ankle jerks, and plantar responses were downgoing.

There were no upper motor neurone or extrapyramidal signs. There was a postural drop in systolic blood pressure (BP) on standing. When assessed 4 months later in addition to the signs of peripheral neuropathy, he had developed mild symmetrical Parkinsonism evidenced by bradykinesia with decrement on repetitive movements, limb rigidity and a jerky rest tremor. Posture was stooped and there was reduced arm swing. He exhibited micrographia. There was also diffuse muscle fasciculation in all four limbs, without wasting. Initial vocal cord assessment was normal but a year after presentation he had developed a paretic right vocal cord. He was commenced on levodopa initially at 50 mg twice a day, increasing to 400 mg/day in four divided doses. A year later, there was no significant improvement nor progression in his Parkinsonism despite the higher dose of levodopa. Although multifactorial, including the sensory ataxic neuropathy, the Parkinsonism and severity of the autonomic symptoms, which progressed to included alternating constipation and diarrhoea, became the major contributors to mobility problems for the patient.

### 2.2 Investigations

Routine blood tests for neuropathy identified an IgG lambda paraprotein with subsequent bone marrow trephine revealing 10% to 15% neoplastic plasma cells without amyloid deposition. Imaging excluded bone lesions and serum amyloid P component (SAP) scan looking for amyloidosis was normal. He was given a concurrent diagnosis of smouldering myeloma. Neurophysiology showed absent upper and lower limb sensory responses, but normal motor action potentials, with a median motor nerve conduction velocity of 50 m/s in keeping with an axonal sensory neuropathy or neuronopathy (Supplementary information). Electromyography was normal. In the absence of motor involvement or conduction slowing, an antibody associated with neuropathy, evidence of amyloid deposition or other features associated with paraproteinaemic neuropathy, it was felt that the paraprotein was an incidental finding. Vestibular assessment revealed normal video head impulse testing, but reduced nystagmic responses to bithermal caloric stimulation bilaterally, suggesting preferential loss of low‐frequency vestibular afferents.

**FIGURE 1:**
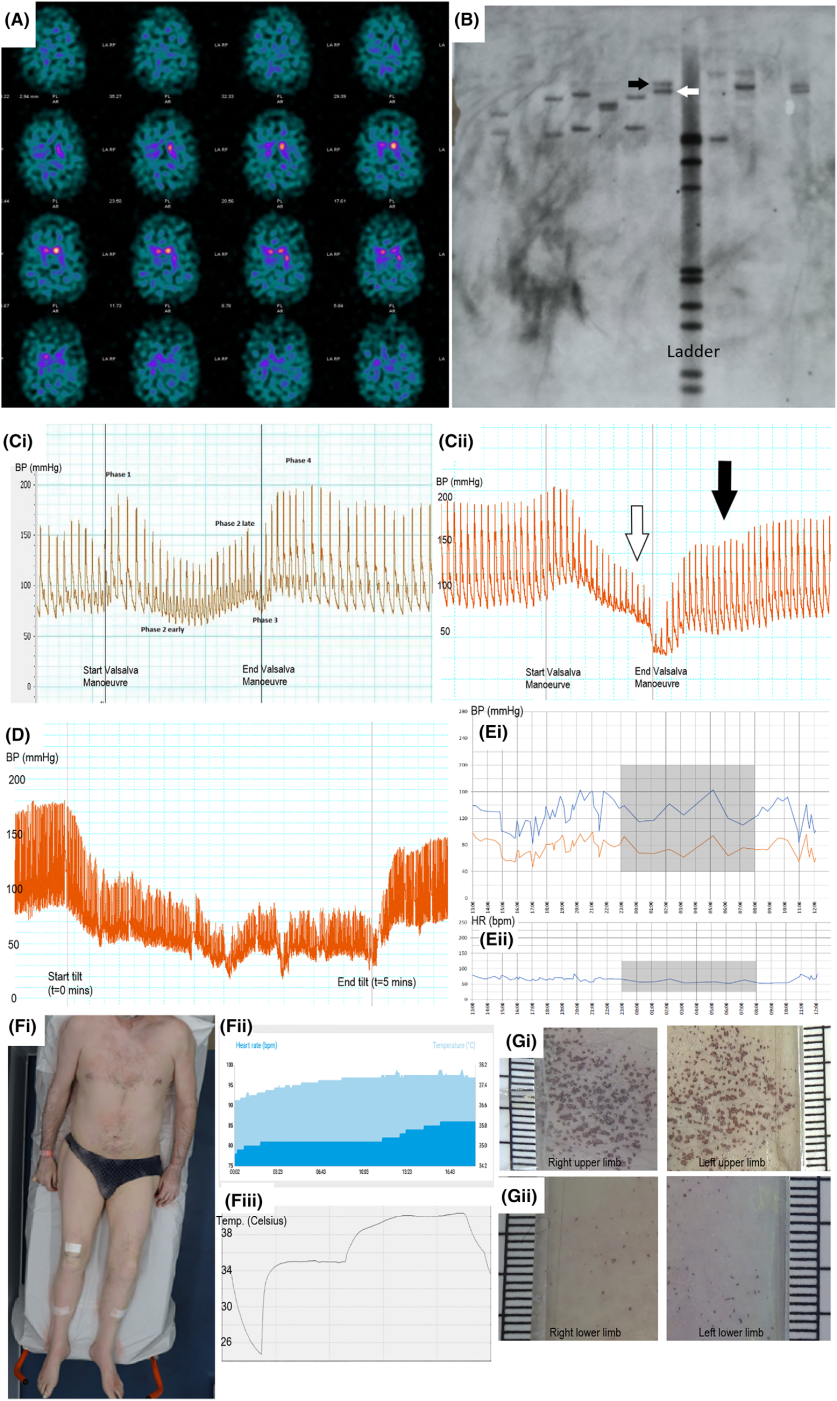
**(**A) DaT scan showing bilaterally markedly diminished basal ganglia uptake. (B) Southern blot of genomic DNA; two bands adjacent to the ladder corresponding to expansion sizes of n = 553 (white arrow) and n = 703 (black arrow), respectively. (C) (i) BP profile showing an example of normal response to Valsalva manoeuvre (ii) abnormal response to Valsalva manoeuvre in our patient; absent phase 2 late recovery (white arrow), and absent phase 4 overshoot (black arrow). (D) BP profile showing severe BP drop over 5 minutes of 60° head up tilt. (E) (i) Ambulatory 24 hours BP monitoring showing evidence of orthostatic hypotension and absent nocturnal circadian fall in BP. Blue line—systolic BP (SBP), orange line—diastolic BP (DBP). (ii) Corresponding heart rate. (F) Thermoregulatory sweat test shows (i) global anhidrosis with (ii) heart rate (lower dark blue trace), skin temperature (upper light blue trace) and (iii) inner ear temperature rise during the test. (G) Dynamic sweat test with (i) almost preserved sweating in upper limbs and (ii) severely reduced sweating in lower limbs

MRI of the brain and spinal cord were unremarkable, including the cerebellum and brainstem. Dopamine transporter (DaT) scan showed bilaterally markedly diminished basal ganglia uptake, more pronounced on the right, consistent with nigrostriatal degeneration (Figure 1A). CT of the neck did not identify a structural cause for the paretic right vocal cord.

Using methods previously described^1^ the patient was found to have biallelic pathogenic intronic AAGGG_(n)_ expansions in *RFC1* with sizes of n = 553 and n = 703 confirmed on southern blotting (Figure 1B).

### 2.3 Autonomic testing

Autonomic testing aged 63 showed orthostatic hypotension (OH) (change in systolic BP, ΔSBP = 35 mmHg at 3 minutes standing and ΔSBP = 52 mmHg at 10 minutes at 60° head‐up tilt), absent heart rate variability to deep breathing and abnormal BP responses to Valsalva manoeuvre (prolonged phase II early, absent phase II late, absent phase IV overshoot and prolonged pressure recovery time, Figure 1C). Catecholamine levels were normal at baseline with only a slight rise on tilt; noradrenaline 207 pg/mL supine and 217 pg/mL on tilt (normal baseline range 200‐500 pg/mL, expected rise >2× baseline). Ambulatory 24‐hour BP profile showed normal nocturnal circadian fall in BP, evidence for OH on standing challenge and no evidence of supine hypertension (not shown). These findings were compatible with widespread sympathetic and parasympathetic cardiovascular autonomic failure. Fludrocortisone 100mcg OD was commenced to control his orthostatic intolerance symptoms.

One year later, the patient reported progressive orthostatic intolerance symptoms with coat hanger pain and exercise intolerance associated with fainting episodes. Autonomic function test on fludrocortisone showed worsening of OH (ΔSBP = 81 mmHg at 3 minutes standing and ΔSBP = 82 mmHg at 5 minutes at 60° head up tilt as he was unable to tolerate 10 minutes tilt, Figure 1D). Both heart rate variability and Valsalva manoeuvre BP profile remained abnormal with reduced Valsalva ratio (1.13 vs 1.34 a year before), 24 hours BP profile showed abnormal circadian BP profile (Figure 1E). Catecholamine levels were normal at baseline with no rise on tilt (noradrenaline 204 pg/mL supine and 204 pg/mL on tilt). Midodrine 2.5 mg tds was prescribed in addition to fludrocortisone.

He underwent thermoregulatory sweat test which showed core temperature increase to 37.8°C and generalised anhidrosis apart from minimal sweating on the dorsum of feet and left wrist (Figure 1F). However, dynamic sweat test showed reduced sweating in length‐dependent pattern with almost preserved sweating in upper limbs (Figure 1G). Cumulatively, these findings were compatible with progressive sympathetic and parasympathetic autonomic dysfunction with pre‐ and post‐ganglionic involvement. Pupillometry was normal as were saliva production and uroflow with no post‐void residual volume.

## 3 INTERPRETATION

This case illustrates the phenotypic variability in patients with biallelic expansions in *RFC1*. Our patient presented with features primarily of sensory neuropathy, in the absence of clear vestibular or cerebellar involvement, associated with autonomic dysfunction and chronic cough, later developing Parkinsonism. Prior to 2019 when *RFC1* was first identified as the causative gene for CANVAS, autonomic failure had been identified as a frequent component of the clinical syndrome and the prevalence of autonomic disturbance in patients with genetically proven *RFC1*‐related disease is reported between 23% and 62%.^1^ However, the autonomic profile of CANVAS/MSA‐like overlap syndrome with confirmed pathogenic biallelic expansion in *RFC1*, had not been fully characterised previously.

The phenotype of autonomic dysfunction in our patient was a widespread autonomic failure of mixed pre‐ and post‐ganglionic involvement with progressive involvement of cardiovascular and sudomotor function. The central preganglionic involvement was evidenced by generalised anhidrosis on TST and normal catecholamine levels at baseline, with minimal rise on head‐up tilt. Peripheral, postganglionic features include abnormal dynamic sweat test in a length‐dependent distribution.

This pattern of mixed pre‐ and postganglionic failure has been seen in patients with multiple system atrophy.^5^ The length‐dependent pattern seen in dynamic sweat test analysis in our patient was also compatible with the predominant pattern of peripheral sudomotor dysfunction in MSA patients.^5^ However, non‐length dependent patterns are also frequently described, and one study showed similarly affected proximal and distal sweat glands, suggesting ganglionopathy rather than length‐dependent pathology in MSA.^6^ The autonomic dysfunction in patients with pure forms of CANVAS is generally less severe compared with MSA.^1^ The neuropathy and vestibular dysfunction in CANVAS is thought to be a ganglionopathy and although pathological studies showing sweat gland denervation have suggested the autonomic pathology is at least in part post‐ganglionic,^7^ the detailed autonomic phenotype has yet to be fully described. Although urinary involvement is more frequently reported in MSA than in CANVAS, a prospective study of MSA patients showed a normal post‐void residual in 25% of cases, so a normal residual volume in our patient is not a helpful distinguishing feature.^8^

In addition, our patient developed a right vocal cord paresis. This is frequently seen in MSA, and is yet to specifically be reported in association with *RFC1*‐related disease. However, there appears to be a subtype of *RFC1*‐multisystem disease with an MSA‐like overlap phenotype, that has early dysphagia compared with its relative absence or late onset in more typical CANVAS,^1^ suggesting a bulbar component to the phenotype. Diffuse fasciculations were also reported in one case of CANVAS family in an Asia‐Pacific family with pathogenic (ACAGG)_n_ expansions,^9^ and recent studies have suggested motor neurone involvement.^10^ The sleep disturbance, although not fully characterised with polysomnography, was suggestive of a REM sleep behaviour disorder. This has been reported in CANVAS,^1^ but is much more commonly seen in MSA.

It remains a possibility that our patient has a dual diagnosis of either idiopathic Parkinsons disease or MSA, plus *RFC1*‐related CANVAS. The lack of response to levodopa makes idiopathic Parkinsons disease less likely. Although the patient could have MSA and CANVAS, the expanding number of reports of an MSA‐like phenotype with *RFC1*‐related disease makes it more likely that all the clinical features in this patient are due to the *RFC1* expansion.^1^, ^2^, ^3^ The fact that there were no cases of biallelic *RFC1* expansions in 336 cases of pathologically confirmed MSA^4^ suggests that *RFC1* expansions are unlikely to cause the neuronal and glial alpha‐synuclein deposition seen in MSA, but rather that *RFC1* causes an MSA‐like syndrome. This should be explored in series of clinically defined, as opposed to pathologically defined, MSA.

In conclusion, we present the first detailed description of the autonomic phenotype as part of *RFC1*‐related multisystem disease, resembling the dysautonomia of MSA. Our case had widespread autonomic failure with pre‐ and post‐ganglionic involvement, with a length‐dependent peripheral pattern demonstrated on dynamic sweat testing, and sympathetic and parasympathetic cardiovascular failure. This case suggests that patients with an MSA‐like syndrome, plus signs of either vestibular failure or sensory neuropathy, should be tested for the *RFC1* repeat expansion.

## AUTHOR CONTRIBUTIONS

Christopher J. Record and Rana Alnasser Alsukhni: study design, acquisition, analysis and interpretation of data and drafting of the manuscript. Riccardo Curro, Diego Kaski, John S. Rubin, Huw R. Morris, Andrea Cortese: acquisition, analysis and interpretation of data and revision of the manuscript. Valeria Iodice and Mary M. Reilly: study design and revision of manuscript.

## FUNDING INFORMATION

Christopher J. Record was supported by an MRC strategic award to establish an International Centre for Genomic Medicine in Neuromuscular Diseases (ICGNMD) MR/S005021/1 and the National Institutes of Neurological Diseases and Stroke and office of Rare Diseases (U54NS065712).

## CONFLICT OF INTEREST

Huw R. Morris is a co‐applicant on a patent application related to C9ORF72—Method for diagnosing a neurodegenerative disease (PCT/GB2012/052140).

## ETHICS STATEMENT

Written consent was obtained from the patient. Participant data were collected in line with the ethically approved study “Charcot‐Marie‐Tooth Disease and related disorders: A Natural History Study,” reviewed by the London Queen Square Research Ethics Committee.

## Supporting information


**Appendix S1:** Supporting Information

## Data Availability

The data that support the findings of this study are available from the corresponding author upon reasonable request.
