# Supplementary material for: Severe distinct dysautonomia in RFC1 ‐related disease associated with Parkinsonism
Source: J Peripher Nerv Syst. 2022 Oct 7;27(4):311–5. doi: 10.1111/jns.12515 (PMC10092280; doi:10.1111/jns.12515)
Supplement: Supplementary file 1 — Appendix S1: Supporting Information [file JNS-27-311-s001.docx]

**Supplementary information**

Neurophysiology; study conducted when patient aged 62 yrs

Sensory nerve conduction studies

|  | Right | Left |
| --- | --- | --- |
| Radial (forearm-snuffbox) | No response | No response |
| Median (d2-wrist) | No response | ND |
| Ulnar (D5-wrist) | No response | ND |
| Sural (calf-ankle) | No response | No response |
| Superf. peroneal (calf-ankle) | No response | No response |

Motor nerve conduction studies

|  |  | Right | | | Left | | |
| --- | --- | --- | --- | --- | --- | --- | --- |
|  |  | Amplitude (mV) | DML (ms) | CV (m/s) | Amplitude (mV) | DML (ms) | CV (m/s) |
| Median (SE on APB) | Wrist | 4.2 | 3.7 | - | ND | - | - |
|  | Elbow | 4.2 | - | 50 | ND | - | - |
| Ulnar (SE on ADM) | Wrist | 9.8 | 3.1 | - | 9.1 | 3.3 | - |
|  | Below elbow | 8.5 | - | 58 | 7.7 | - | 58 |
|  | Above elbow | 7.9 | - | 55 | 7.5 | - | 60 |
| Common peroneal (SE on EDB) | Ankle | 2.5 | 4.3 | - | 1.3 | 4.8 | - |
|  | Fib neck | 2.3 | - | 36 | 1.5 | - | 39 |
| Tibial (SE on AH) | Ankle | 6.5 | 6.1 | - | 4.9 | 6.1 | - |
|  | Pop fossa | 3.4 | - | 40 | 3.5 | - | 40 |

ND = not done, SE = sensory electrode, DML = distal motor latency, CV = conduction velocity, APB = abductor pollicis brevis, ADM = abductor digiti minimi, EDB extensor digitorum brevis, AH = Abductor Hallucis
